# Strain assisted electrocaloric effect in PbZr_0.95_Ti_0.05_O_3_ films on 0.7Pb(Mg_1/3_Nb_2/3_)O_3_-0.3PbTiO_3_ substrate

**DOI:** 10.1038/srep16164

**Published:** 2015-11-04

**Authors:** Zhenghu Zuo, Bin Chen, Baomin Wang, Huali Yang, Qingfeng Zhan, Yiwei Liu, Junling Wang, Run-Wei Li

**Affiliations:** 1 Key Laboratory of Magnetic Materials and Devices, Ningbo Institute of Materials Technology and Engineering, Chinese Academy of Sciences, Ningbo, Zhejiang 315201, China; 2 Zhejiang Province Key Laboratory of Magnetic Materials and Application Technology, Ningbo Institute of Materials Technology and Engineering, Chinese Academy of Sciences, Ningbo 315201, China; 3 School of Materials Science and Engineering, Nanyang Technological University, Singapore 639798, Singapore

## Abstract

Solid state cooling technologies based on electrocaloric, magnetocaloric and mechanocaloric effects have received much attention during the past decade. To further improve the cooling efficiency and reduce the driving field, it is desirable to combine multiple effects in a single system. Here, we report on the caloric effects induced by both electric field and strain in PbZr_0.95_Ti_0.05_O_3_ films deposited on 0.7Pb(Mg_1/3_Nb_2/3_)O_3_-0.3PbTiO_3_ substrate. The isothermal entropy change (*ΔS*) induced by the antiferroelectric-ferroelectric phase transition of PbZr_0.95_Ti_0.05_O_3_ films is calculated to be 6.78 J K^−1^ kg^−1^. Furthermore, the strain from 0.7Pb(Mg_1/3_Nb_2/3_)O_3_-0.3PbTiO_3_ substrate can reduce the electric field where *ΔS* reaches the maximum by as much as 50 kV/cm. The electrocaloric efficiency is also increased from 0.366 to 0.378 by the strain effect. The electrocaloric effect in an antiferroelectric material assisted by strain may lead to more efficient solid state cooling technology.

The search for a highly efficient and environmentally friendly solid state cooling technology has attracted much attention recently^1^,^2^,^3^,^4^,^5^,^6^,^7^,^8^,^9^. It offers an alternative to the traditional vapor-compression cooling method widely used in household and industrial applications, which involves hazardous gases. Several technologies that are currently being developed make use of various caloric effects, i.e. electrocaloric (EC), magnetocaloric (MC), and/or mechanocaloric (mC) effect. Caloric effect refers to a change in temperature under adiabatic application of external fields. Alternatively, it can be described as an isothermal change in entropy. To achieve a large caloric effect, a phase transition is desirable where the entropy change (*ΔS*) is maximized. For example, the giant mC, MC, and EC effects observed in the shape memory alloy Cu_69.6_Al_27.7_Ni_2.7_^7^, the metallic Fe_49_Rh_51_^8^, and the ceramic PbZr_0.95_Ti_0.05_O_3_ (PZT)^9^ are associated with martensite-austenite, antiferromagnetic-ferromagnetic, and ferroelectric-paraelectric phase transitions, respectively. Each of these caloric effects is induced by a particular driving field. Moving forward, it is natural to investigate caloric effects driven by multi-fields for better performance, as proposed by Moya *et al.* in a recent review^1^.

It is well known that the ferroic phase transition usually leads to a large change in entropy^2^. In 2006, large EC effect was observed experimentally in ferroelectric PZT^9^. Recently, a theoretical study has shown that giant mC can be expected in ferroelectric Ba_0.5_Sr_0.5_TiO_3_^10^ and BaTiO_3_^11^. However, there are few experimental reports on caloric effects driven by multi-fields up to now^12^. In this paper, we report the caloric effect driven by both electric field and strain in antiferroelectric PZT films on 0.7Pb(Mg_1/3_Nb_2/3_)O_3_-0.3PbTiO_3_ (PMN-PT) substrate. The idea is that both the electric field applied to the films and the strain from the substrate can contribute to the *ΔS* of the PZT films. Indeed, a large *ΔS* of about 6.78 J K^−1^ kg^−1^ can be obtained in the hybrid system.

## Results

**Figure 1:**
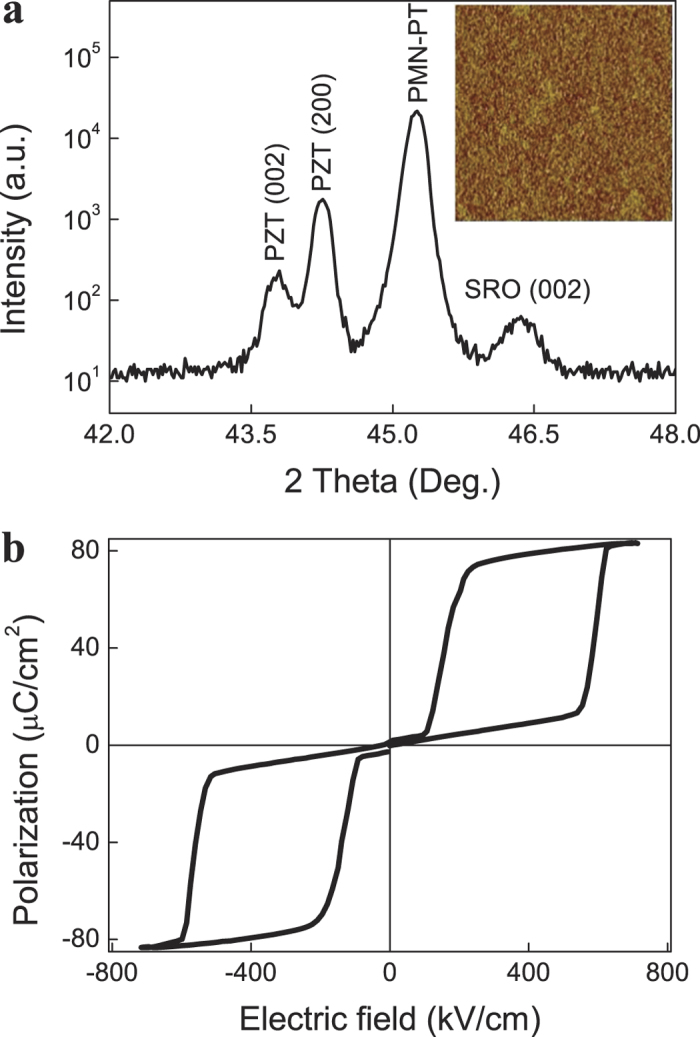
The structure and P-E loop of PZT on PMN-PT at room temperature. (**a**) XRD pattern and AFM image (inset, 10 × 10 μm^2^) of the PZT film on PMN-PT substrate. (**b**) Typical *P-E* loop of the PZT films at room temperature.

300 nm PZT films were prepared on (001) orientation PMN-PT substrates with 30 nm SrRuO_3_ (SRO) bottom electrode by pulsed laser deposition (PLD). Figure 1a shows the XRD *θ*–2*θ* pattern of the sample. In addition to the pseudo-cubic (200) peaks of the substrate and SRO layer, only peaks from the (200) and (002) planes of the tetragonal PZT can be seen, which demonstrates that 90^o^ *a-c* domains are present in the film. It can also be seen that the films are *a*-domain dominated. The AFM image of the PZT film is displayed in the inset of Fig. 1a, which shows that the roughness is very small (~3 nm). The electric field dependence of polarization (*P-E* loop) of the PZT films at room temperature is shown in Fig. 1b, typical of an antiferroelectric material.

**Figure 2:**
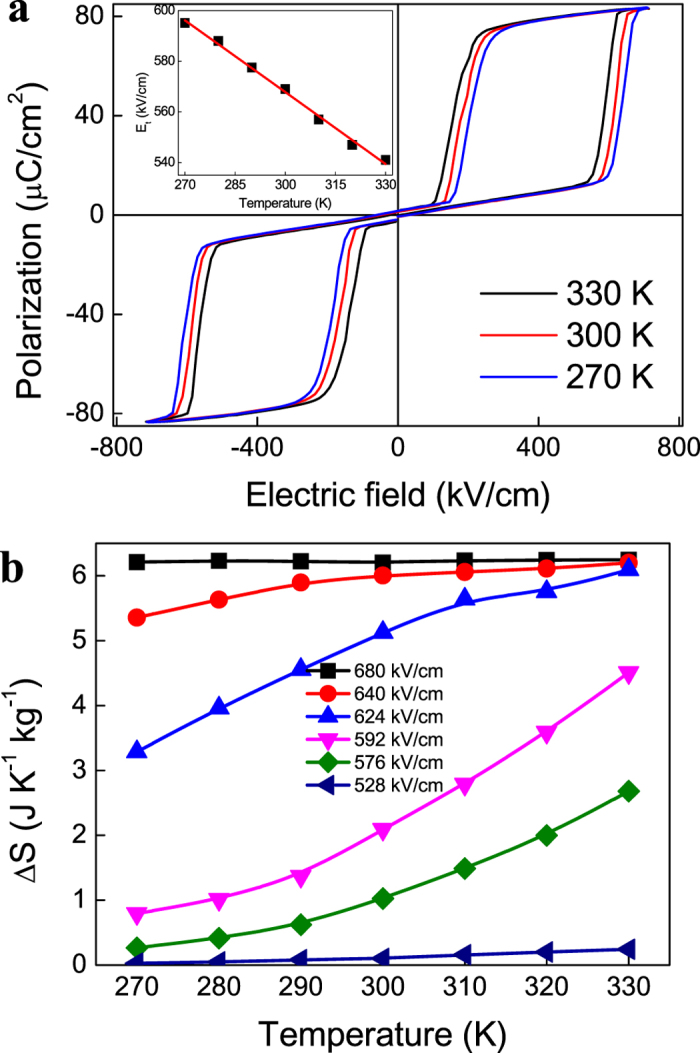
Temperature dependences of *P-E* loops and *ΔS* of PZT on PMN-PT at different electric fields. (**a**) *P-E* loops at selected temperatures. The inset shows the transition field as a function of temperature. The line is a linear fit *E*_*t*_ = −0.94*T* + 851. (**b**) Electric field induced *ΔS* at selected values of electric field ranging from 528 to 680 kV/cm.

The caloric effect of meta-magnetic materials at the magnetic transition has been reported extensively^1^,^13^. Similarly, *ΔS* also occurs at the antiferroelectric-ferroelectric transition in antiferroelectric materials. In order to evaluate the caloric effect in the antiferroelectric PZT films, the *P-E* loops are measured at various temperatures between 270 K and 330 K and selected results are shown in Fig. 2a. From the *P-E* loops we can define a transition electric field, *E*_*t*_, at each temperature. The polarization change, 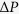, at the transition can also be estimated from these curves. In the inset of Fig. 2a we show the transition field as a function of temperature. We see that *E*_*t*_ rises linearly with decreasing *T*, with a slope of 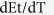
= −0.94 kV/cm K.

The *ΔS* due to first-order phase transitions in magnetic materials and shape-memory alloys have been calculated by using the modified Maxwell functions^6^,^14^. Assuming a system subjected to an electric field *E* undergoes a first-order phase transition in equilibrium at a temperature *T*_*t*_, the transition is characterized by discontinuities in variables such as polarization and entropy that are thermodynamically conjugated to the electric field and temperature. In the vicinity of the transition, the following behavior of the polarization is thus expected, *P(T, E) = P*_*0*_* + ΔP *F* [(T*_*t*_ *(E)-T)/ΔT]*, where *F* is a shape-function and *ΔT* is the temperature range over which the transition spreads. In strict equilibrium, *ΔT*
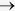
0 and *F* approaches the Heaviside step function. Following the expression 
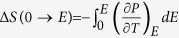
 and assuming that *P*_*0*_ and *ΔP* are constants, the caloric effect in the vicinity of the transition in this equilibrium case is given by


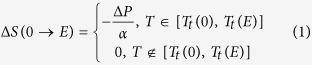


where 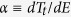 is assumed to be constant. Taking into account the Clausius-Clapeyron equation, 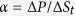, where 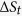
is the transition entropy change. Therefore, as expected 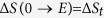, and 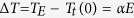. The caloric effect (electric field-induced *ΔS*) has been obtained by numerically computing the integral in *Eq.* (1). The result is shown in Fig. 2b. Note that the maximum *ΔS* of 6.78 J K^−1^ kg^−1^ remains almost constant (thus independent of temperature and applied stress) over a very broad temperature range.

**Figure 3:**
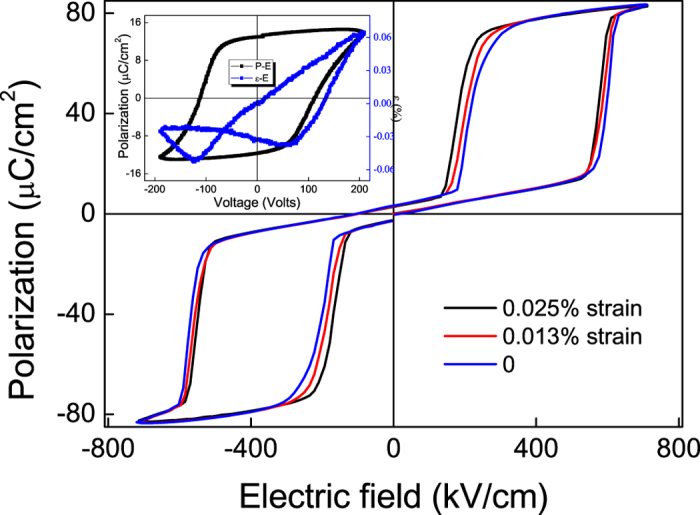
*P-E* loops of PZT on PMN-PT at 330 K under different applied strains. The inset shows the electric field dependence of polarization and strain of PMN-PT substrate.

It is well known that the antiferroelectric-ferroelectric phase transition in antiferroelectric materials is usually accompanied by a large volume change^15^. During the phase transition, the lattice parameter *a* decreases while *c* increases. Therefore, if we apply a tensile strain in the (001) plane of the antiferroelectric films, *E*_*t*_ will increase. Conversely, the opposite occurs if we apply a compressive strain. Using PMN-PT as the substrate, we can apply electric field on the substrate to induce an in-plane tensile strain to the film. The tensile strain will reduce the *E*_*t*_ of PZT films because our samples are *a*-domain dominated as discussed above. The electric field dependence of polarization and strain of the PMN-PT substrate is shown in the inset of Fig. 3. The shift of loops is due to the asymmetric electrodes used. When the electrode generates an out-of-plane compressive strain of −0.05% (@ 4 kV/cm), an in-plane tensile strain of 0.025% is induced. *P-E* loops of the PZT films at 330 K under different strain states are shown in Fig. 3, from which we can see that the transition field of the PZT film shifts to a lower value when the strain becomes larger while the saturation polarization remains almost the same.

**Figure 4:**
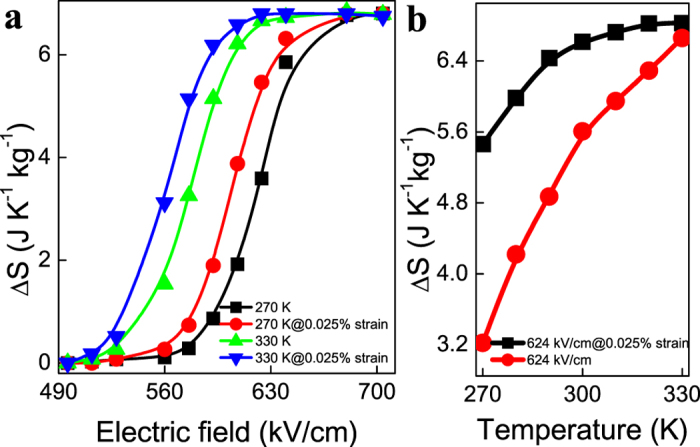
Electric field and temperature dependence of *ΔS* under different strains applied from PMN-PT substrate. (**a**) Electric field dependence of *ΔS* at two selected temperatures with and without applied strain. (**b**) Temperature dependence of *ΔS* at 624 kV/cm with different strain applied.

The electric field and strain induced *ΔS* at selected temperatures is shown in Fig. 4. We can see that when the electric field applied to PZT is lower than 512 kV/cm, the antiferroelectric-ferroelectric transition cannot occur. Therefore, the *ΔS* is zero. When the electric field is larger than 688 kV/cm, the transition is completed. As a result, the *ΔS* reach a maximum value. Compared to the transition caused by electric field only, the total *ΔS* induced by the combination of electric field and strain remains relatively unchanged. However, the strain reduces the electric field needed to achieve certain *ΔS*. For PZT films at 330 K, the electric field where *ΔS* reaches the maximum is reduced by ~ 50 kV/cm due to strain from the substrate. When we apply an electric field of 624 kV/cm to the PZT films, the *ΔS* at 270 K is about 3.2 J K^−1^ kg^−1^ while the *ΔS* under a 0.025% strain is around 5.5 J K^−1^ kg^−1^ (shown in Fig. 4b). At 330 K, the difference between different strain states is very small. Therefore, when a certain electric field lower than *E*_*t*_ is applied to the PZT films, external strain can result in larger *ΔS* over a wide temperature range. We have also considered the electrocaloric efficiency (*η*) of the hybrid system as introduced by Defay *et al.*^16^, which equals to the ratio of reversible electrocaloric heat (*Q*) to reversible electrocaloric work (*W*) under isothermal condition. The value of *Q* can be evaluated following 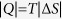, while *W* can be calculated following 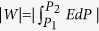. The calculated *η* is about 0.366 for the PZT film alone while it increases to 0.378 when −0.05% strain induced by PMN-PT is applied to the PZT film.

## Discussion

For entropy change for ferroelectric to paraelectric transition, a large value could be only obtained around the Curie temperature (*T*_*c*_). When the work temperature is away from the *T*_*c*_, the entropy change will be very small. Therefore, the work temperature of the cooling device using ferroelectric to paraelectric transition was limited to the *T*_*c*_ of the functional material. However, a relatively large value of the entropy change for antiferroelectric to ferroelectric transition could be arrived over a wide range only if the work temperature is lower than the *T*_*c*_ of the functional material. Our findings are especially useful for bulk ferroelectric ceramic samples because they usually cannot withstand the high electric field that thin films can. With a pre-load strain, a lower electric field can result in larger *ΔS*, ideal for electrocaloric solid-state refrigeration technology^17^.

In conclusion, a large *ΔS* of 6.78 J K^−1^ kg^−1^ is obtained from the antiferroelectric-ferroelectric phase transition in PZT films induced by electric field and strain from the substrate. The strain helps to reduce the electric field needed to achieve maximum *ΔS* by as much as 50 kV/cm and increases the electrocaloric efficiency from 0.366 to 0.378. Furthermore, *ΔS* can be obtained in a broad range of temperatures which opens up interesting opportunities in refrigeration applications based on the caloric effect.

## Methods

### Specimen Fabricatio**n**

Pulsed laser deposition (PLD) was used to prepare the SrRuO_3_ (SRO) bottom electrode and PZT film on (001) orientation PMN-PT substrate at 800 °C with an oxygen partial pressure of 10 Pa. After the deposition of PZT film, 100 nm Cu top electrodes of 100 × 100 μm^2^ were grown by thermal evaporation using a metal mask.

### Characterization

The surface morphologies of the as-grown films were studied using an atomic force microscopy (Dimension3100V, Bruker). The structure of the films were characterized using an X-ray diffraction (D8 Advance, Bruker). Ferroelectric properties were measured using a commercial ferroelectric tester (Precision Premier II, Radiant Technology).

## Additional Information

**How to cite this article**: Zuo, Z. *et al.* Strain assisted electrocaloric effect in PbZr_0.95_Ti_0.05_O_3_ films on 0.7Pb(Mg_1/3_Nb_2/3_)O_3_-0.3PbTiO_3_ substrate. *Sci. Rep.* **5**, 16164; doi: 10.1038/srep16164 (2015).
